# Avaliação do impacto da implantação do novo sistema de informações da atenção primária à saúde nos registros de atendimentos e visitas domiciliares no Brasil

**DOI:** 10.1590/0102-311XPT081323

**Published:** 2024-01-08

**Authors:** Rafael Damasceno de Barros, Livia Angeli Silva, Luis Eugenio Portela Fernandes de Souza

**Affiliations:** 1 Universidade Federal da Bahia, Salvador, Brasil.

**Keywords:** Atenção Primária à Saúde, Sistema de Informação em Saúde, Avaliação do Impacto na Saúde, Primary Health Care, Health Information Systems, Health Impact Assessment, Atención Primaria de Salud, Sistemas de Información en Salud, Evaluación del Impacto en la Salud

## Abstract

A substituição do Sistema de Informação da Atenção Básica (SIAB, 1998-2015), a partir de janeiro de 2016, pelo novo Sistema de Informação em Saúde para a Atenção Básica (SISAB) determinou novas formas de coleta, processamento e uso das informações, com possível impacto nos registros das atividades desenvolvidas na atenção primária à saúde no Brasil. O objetivo deste estudo foi avaliar o impacto da implantação do novo sistema de informação sobre registros de atendimentos de médicos e enfermeiros, e de visitas domiciliares de agentes comunitários de saúde (ACS) brasileiros entre 2007 e 2019. Para tal, utilizou-se uma abordagem bayesiana de modelo estrutural para séries temporais, com base em uma regressão difusa de espaço-estado. Ao longo do período de 2016 a 2019, foram registrados 463,47 milhões de atendimentos médicos, 210,61 milhões de atendimentos de enfermagem e 1,28 bilhão de visitas de ACS. Seguindo a tendência registrada antes da implantação, seriam esperados 598,86 milhões, 430,46 milhões e 1,5 bilhão de atendimentos de médicos, enfermeiros e visitas de ACS, respectivamente. Em termos relativos, houve um decréscimo de 25% nos atendimentos médicos, 51% nos atendimentos de enfermagem e 15% nas visitas de ACS quando comparado com o valor esperado pelo método bayesiano. O impacto negativo no registro de atendimentos e de visitas domiciliares identificado neste estudo, seja por dificuldade de adaptação ao novo sistema, seja por diminuição de registros indevidos, merece ser alvo de investigação para que se possa, de forma planejada, compreender e superar o desafio da melhoria do sistema de informação da atenção primária.

## Abstract (en)

The replacement of the Primary Care Information System (SIAB, 1998-2015), as of January 2016, by the new Health Information System for Primary Care (SISAB) determined new forms of collecting, processing, and using information, with a possible impact on the records of activities carried out in primary health care in Brazil. This study aimed to evaluate the implementation impact of the new information system on records of physicians’ and nurses’ patient care and home visits of community health workers (CHW) in Brazil from 2007 to 2019. To this end, a Bayesian structural time-series model approach was used, based on a diffuse state-space regression. From 2016 to 2019, 463.47 million physician care, 210.61 million nursing care, and 1.28 billion CHW visits were recorded. Following the trend recorded before the implementation, 598.86 million, 430.46 million, and 1.5 billion physician and nursing appointments and CHW visits would be expected, respectively. In relative terms, there was a decrease of 25% in physician care, 51% in nursing care, and 15% in CHW visits when compared to the value expected by the Bayesian method. The negative impact on the records of patient care and home visits identified in this study, whether due to difficulties in adapting to the new system or a reduction in improper records, must be investigated so that the challenge of improving the primary care information system can be understood and overcome in a planned way.

## Resumen

La sustitución del Sistema de Información de la Atención Básica (SIAB, 1998-2015), desde enero de 2016, por el nuevo Sistema de Información en Salud para la Atención Básica (SISAB) estableció nuevas maneras para recolectar, procesar y utilizar las informaciones, con posibles impactos en los registros de las actividades desarrolladas en la atención primaria de salud en Brasil. El objetivo de este estudio fue evaluar el impacto de la implantación del nuevo sistema de información sobre los registros de atención de médicos y enfermeros y de visitas domiciliarias de agentes comunitarios de salud (ACS) en Brasil entre 2007 y 2019. Para eso, se utilizó un enfoque bayesiano de modelo estructural para series temporales basadas en una regresión difusa de espacio de estado. Entre los años 2016 y 2019, se registraron 463,47 millones de consultas médicas, 210,61 millones de consultas de enfermería y 1,28 mil millones de visitas de ACS. Siguiendo la tendencia registrada antes de la implantación, se esperarían 598,86 millones, 430,46 millones y 1,5 mil millones de consultas médicas y de enfermería y visitas de ACS respectivamente. En términos relativos, hubo una disminución del 25% en las consultas médicas, del 51% en las consultas de enfermería y del 15% en las visitas de ACS en comparación con el valor esperado por el método bayesiano. El impacto negativo en el registro de consultas y visitas domiciliarias identificado en este estudio, ya sea por dificultades en la adaptación al nuevo sistema o por la disminución de los registros indebidos, merece ser objeto de investigación para que se pueda, de manera planificada, comprender y superar el desafío continuo de mejorar el sistema de información de la atención primaria.

## Introdução

A atenção primária à saúde (APS) é reconhecida como uma das estratégias mais efetivas na redução de mortes e internações por vários agravos e doenças, principalmente doenças crônicas não transmissíveis ^1^^,^^2^^,^^3^^,^^4^^,^^5^^,^^6^. Desde a década de 1990, a APS vem sendo desenvolvida em larga escala no Brasil e, em 2019, já estava em 99,7% dos municípios ^7^.

Em 2019, aproximadamente 156 milhões de pessoas eram atendidas pela APS (74,76% da população brasileira). Nesse ano, foram registrados mais de 136 milhões de atendimentos médicos, 66 milhões de atendimentos de enfermagem e 329 milhões de visitas domiciliares de agentes comunitários de saúde (ACS) ^8^.

Desde a implantação da APS no âmbito do Sistema Único de Saúde (SUS), numa perspectiva territorial, o Ministério da Saúde tem buscado formas de sistematizar informações desse primeiro nível de atenção sobre as condições de saúde, o perfil sociodemográfico e os serviços prestados. Para tanto, foi implantado, em janeiro de 1998, o Sistema de Informação da Atenção Básica (SIAB) que, sem dúvida, contribuiu para o avanço da APS no Brasil ^9^.

Após alguns anos, contudo, ficaram evidentes as limitações do SIAB, que, por exemplo, não permitia identificar os usuários, nem codificar todas as doenças ^10^. Ademais, o SIAB se baseava no registro manual de dados, seja por dificuldades de provisão de equipamentos de informática, seja pela insuficiente capacitação de recursos humanos.

O SIAB funcionava por meio do preenchimento de fichas com marcadores de produção diários (p.ex.: quantidade de consultas de médicos e enfermeiros), que deveriam ser somadas, consolidadas e enviadas mensalmente para a Secretaria Municipal de Saúde, responsável pela digitação dos dados no sistema. Essa consolidação dos dados poderia ocorrer de diferentes formas, seja pelo próprio profissional que realizou os atendimentos ou outro designado para tal função. Esse processo heterogêneo de consolidação de fichas e posterior envio era desafiador para o processo de gestão da APS e gerava potencial risco de erros no registro da quantidade dos atendimentos. Parte dessas dificuldades pareciam passíveis de superação em 2013 ^11^.

Por essas razões, o Ministério da Saúde decidiu implantar o novo Sistema de Informações em Saúde da Atenção Básica (SISAB), dentro de uma estratégia de informatização da atenção básica do SUS (e-SUS AB). O SISAB teve sua operação iniciada em abril de 2013, funcionando em concomitância com o SIAB até dezembro de 2015. Em janeiro de 2016, o SISAB passa a ser o único sistema de informação disponibilizado pelo Ministério da Saúde para registro das atividades da APS no Brasil.

Segundo Cielo et al. ^12^, o novo sistema, informatizando a APS, favorece a adoção do prontuário eletrônico, apoio à gestão do cuidado, otimização da coleta de dados, diálogo com os outros sistemas utilizados pela APS e o detalhamento das informações de saúde por meio da individualização dos registros, rompendo a lógica de dados consolidados utilizada na APS até então.

O processo de registro dos atendimentos ainda era passível de erros, porém, com a introdução de campos obrigatórios que identificavam o usuário (como o número do Cartão Nacional de Saúde), criou-se uma certa camada de proteção, em que atendimentos com dados obrigatórios não preenchidos ou não cadastrados nas bases dos sistemas do Ministério da Saúde eram devolvidos e não contabilizados pelo SISAB, oportunizando os profissionais a corrigir tais erros, para que o atendimento fosse devidamente contabilizado. Assim, o registro dos processos no novo sistema era consideravelmente diferente do modelo utilizado no SIAB.

De acordo com a teoria da mudança ^13^, na saúde, a alteração de processos é desafiadora, diante da complexidade do setor, exigindo das organizações condições especiais de gerenciamento ^14^. A implantação do novo SISAB provocou modificações tanto nas formas de coleta, processamento e uso das informações, quanto no processo de cuidado e gestão dos serviços de saúde como um todo ^15^.

Na prática, a partir de 2016, observa-se uma redução nos números de atendimentos da APS ^7^^,^^8^, levantando-se a hipótese de que não era uma redução da prestação de serviços à população, mas sim uma diminuição do registro dos atendimentos realizados, decorrente da substituição dos sistemas de informação.

Diante dessa hipótese, o objetivo deste estudo foi avaliar o impacto da implantação do SISAB sobre os registros de atendimentos médicos e de enfermagem e visitas domiciliares de ACS nas APS dos municípios brasileiros.

## Métodos

Para a avaliar o impacto da implantação do novo SISAB nos registros de atividades da APS, foi utilizada uma abordagem bayesiana de modelo estrutural para séries temporais ^16^. Tal método propõe inferir o impacto causal com base em um modelo de regressão difusa de espaço-estado que prevê a resposta contrafactual dos tratados a partir de um controle sintético que expressa o que teria ocorrido se não tivesse havido intervenção. Em contraste com os esquemas clássicos de diferenças em diferenças, os modelos de espaço-estado permitem: (a) inferir a evolução temporal do impacto atribuível; (b) incorporar características empíricas anteriores aos parâmetros em um tratamento totalmente bayesiano; e (c) de forma flexível, acomodar múltiplas fontes de variação, incluindo tendências locais, sazonalidade e a influência variável no tempo de covariáveis contemporâneas, utilizando um algoritmo de Monte Carlo via Cadeia de Markov (MCMC) para inferência posterior. A análise foi realizada por meio do pacote *CausalImpact* do software R, v. 4.1.2 (http://www.r-project.org), construído pelos autores do modelo ^16^.

O modelo ideal de análise de impacto seria realizado a partir de experimento controlado com uma seleção aleatória de municípios onde o SISAB é o único sistema de informações, constituindo o grupo de tratados e uma outra seleção aleatória de municípios que continuariam utilizando o SIAB, comparando-se a diferença na média da variável resposta. Tal nível de controle sobre a implementação de políticas públicas, em geral, é raro, e, no caso em estudo, é inexistente.

O SISAB foi disponibilizado pelo Ministério da Saúde e seu uso passou a ser obrigatório em todo o território nacional, impossibilitando a constituição de um grupo de tratados e controles sem a intervenção. Diante dessa limitação, o modelo aplicado de avaliação de impacto ^16^ propõe, entre outros parâmetros, a construção de um controle sintético a partir de covariáveis que têm forte correlação com a variável resposta e não foram afetadas pela intervenção. Para tal, o modelo exige duas suposições: as séries temporais de controle não foram afetadas pela intervenção; e a correlação entre as covariáveis e a variável resposta permaneceu estável em todo o período (antes e após a intervenção).

O impacto da implantação do SISAB foi avaliado a partir dos registros de três variáveis respostas: atendimentos individuais de médicos, atendimentos individuais de enfermeiros e visitas domiciliares de ACS. Para construir o controle sintético, foram utilizados a estimativa do percentual de população coberta pela APS e, para cada caso, o número de profissionais na APS (médicos, enfermeiros e ACS).

Assumiu-se, neste trabalho, que o fim do funcionamento do SIAB, com o registro das atividades passando a ser obrigatório no SISAB, não tem nenhuma relação com a equipe de profissionais da APS, já que não há nenhuma evidência de que a mudança do sistema de informação tenha relação com a alocação dos recursos humanos nas unidades de saúde. Contudo, parte-se do pressuposto de que o número de atendimentos individuais e visitas domiciliares é uma função do número de profissionais e do percentual de população coberta, ou seja, a expansão da cobertura, potencialmente, gerará aumento do número total de atendimentos e visitas. Tampouco há evidências de que essa relação tenha mudado dentro do período analisado.

Para confirmar tais assunções, foi utilizado o modelo de análise de séries temporais interrompidas ^17^ que adota uma abordagem centrada na variância para estimar a mudança em uma variável resposta ao longo do tempo. Foi utilizado um modelo de análise de covariância (ANCOVA) de variável dependente defasada de tipo 2 (*Type II Sum Squares ANCOVA Lagged Dependent Variable*) para avaliar se as variáveis escolhidas se comportam como controles adequados para a análise de impacto da intervenção.

Considerando os mesmos períodos de tratados e controles, não foi encontrada variação significativa tanto para a estimativa de população coberta (valor de p = 0,49), número de médicos (valor de p = 0,47), número de enfermeiros (valor de p = 0,52) e ACS (valor de p = 0,15), o que indica que a mudança do sistema de informação não teve relação com a estrutura de recursos humanos da APS.

A confirmação de que não houve variação no número de profissionais e na cobertura populacional da APS, antes e depois da intervenção, reforça a hipótese de que a redução no número de atividades registradas decorreu de dificuldades no registro e não de uma real diminuição da realização de atendimentos e visitas.

Utilizar a cobertura da APS, além do número de profissionais, como covariável adiciona algumas camadas à avaliação, já que a ampliação do número de profissionais pode não resultar no aumento da cobertura diante das diferentes cargas horárias de cada profissional na APS e do crescimento demográfico da população, características levadas em conta pelo Ministério da Saúde no cálculo da estimativa de população coberta pela APS.

Foi calculado o efeito pontual (mensal) e acumulado ao longo do período pós-intervenção, considerando a diferença entre a série temporal observada e a estimada pelo modelo que prevê o comportamento da variável resposta na ausência da intervenção, baseada no seu comportamento antes da intervenção, no controle sintético, na tendência a nível local, no efeito do tempo sobre as covariáveis e na sazonalidade.

Os atendimentos individuais de médicos e enfermeiros e as visitas domiciliares de ACS são atividades registradas tanto no SIAB como no SISAB, e perfazem juntos, em média, 79% de todas as atividades passíveis de registros na APS.

Em 2007, o Ministério da Saúde definiu nova fórmula de cálculo da estimativa de cobertura da APS, estando os dados disponíveis a partir de agosto. Portanto, esse mês foi tomado como ponto inicial do período a ser avaliado neste estudo. Os dados de cobertura e número de ACS foram coletados no portal e-Gestor Atenção Básica do Ministério da Saúde (https://egestorab.saude.gov.br/), por meio do relatório de acesso público. Os quantitativos de médicos e enfermeiros da APS foram coletados no sistema de Cadastro Nacional de Estabelecimentos de Saúde (CNES), considerando os profissionais médicos e enfermeiros de todas as especialidades que atendem no SUS no nível de complexidade atenção básica, atuando em centro de saúde/unidade básica de saúde, posto de saúde, unidade de saúde da família ou unidade móvel fluvial. Os dados de atendimentos e visitas, referentes ao período de agosto de 2007 a dezembro de 2015, foram coletados no SIAB por meio da página do Departamento de Informática do SUS (Datasus; http://www2.datasus.gov.br/SIAB/index.php). Os dados do SISAB foram coletados entre abril de 2013 a dezembro de 2019, em página de internet (https://sisab.saude.gov.br/) disponibilizada pelo Ministério da Saúde.

Entre abril de 2013 e dezembro de 2015, diante da possibilidade do uso dos dois sistemas, os dados de atendimentos médicos, de enfermeiros e visitas domiciliares de ACS foram somados, partindo do pressuposto que a implementação do SISAB foi heterogênea ocorrendo em momentos diferentes em cada município. Esse cenário impõe uma limitação para os achados deste trabalho, que focou sua análise no nível nacional. Portanto, o impacto médio calculado no nível do país não pode ser extrapolado ao nível do município, já que o processo de implementação ocorreu em períodos e velocidades diferentes em cada ente federativo. Como nacionalmente o uso único do SISAB ocorreu a partir de janeiro de 2016, este estudo considera o impacto da implementação desde essa data; contudo, o impacto da implementação em cada município pode ter ocorrido quando decidiram utilizar o SISAB, até mesmo antes de janeiro de 2016.

Evitou-se utilizar dados de 2020 e 2021 diante do contexto da pandemia de COVID-19 que influenciou o acesso e a provisão de serviços na APS brasileira ^18,^ ^19^.

Os dados das três variáveis respostas têm autocorrelação serial, tendência linear local e sazonalidade. O modelo de regressão difusa de espaço-estado considera todas essas características, para evitar um viés que poderia resultar em superestimação. Os *a priori* do modelo são calculados utilizando as variáveis preditoras de controles (cobertura da APS e número de profissionais), considerando a média, desvio padrão (DP) e número de observações de cada preditor para calcular o peso no modelo de regressão, assim como a fração da variância explicada da variável dependente (número de atendimentos ou visitas domiciliares) por cada variável preditora e o DP da variável dependente, o que reforça a necessidade de que tais variáveis estejam correlacionadas com a variável dependente, mas não com a intervenção. Portanto, foi utilizado neste estudo um *a priori* que utiliza um componente de tendência de nível local considerando uma distribuição gama inversa com DP = 0,01, diante da estabilidade das variáveis preditoras ao longo do tempo, indicando estabilidade dos resíduos no período pré-intervenção das frações de explicação das variáveis preditoras sobre a variância da variável dependente.

O segundo componente considerou os parâmetros da regressão estática das covariáveis selecionadas pelo próprio algoritmo bayesiano do modelo. Foi utilizado, também, um componente de sazonalidade de 12 meses e 1.000 repetições nas amostras do modelo de MCMC.

## Resultados

Foi analisada a produção de serviços registrada nos sistemas de informação da APS brasileira por todos os municípios em 149 meses (de agosto de 2007 até dezembro de 2019). A Tabela 1 apresenta um descritivo das variáveis resposta e de controle. No período, houve, em média mensal, mais atendimentos médicos (10,53 milhões) do que de enfermagem (6,54 milhões). Já os ACS realizaram, em média mensal, 28,06 milhões de visitas domiciliares. Especificamente em 2019, houve, em média mensal, 11,4 milhões de atendimentos médicos, 5,5 milhões de atendimentos de enfermagem e 27,5 milhões de visitas domiciliares de ACS.

**Tabela 1:** Descritivo das variáveis por mês, antes e depois da implementação obrigatória do Sistema de Informação em Saúde para a Atenção Básica (SISAB), Brasil.

Variáveis	Número de meses	Média	Mediana	DP	Mínimo	Máximo
Antes da implementação *						
Variáveis resposta						
Atendimentos médicos **	101	10,9	10,9	1,28	7,78	14,1
Atendimentos de enfermagem **	101	7,56	7,56	1,04	5,08	11,0
Visitas domiciliares de ACS ***	101	28,9	29,0	1,74	24,5	36,2
Variáveis de controle						
Estimativa do % de cobertura da APS	101	69,52	69,29	4,52	58,48	75,37
Número de médicos(as) **	101	41,0	38,9	4,64	37,23	50,2
Número de enfermeiros(as) *	101	45,8	45,4	4,91	35,87	53,9
Número de ACS ^#^	101	265,0	266,8	14,6	228,2	282,1
Depois da implementação ^#^						
Variáveis resposta						
Atendimentos médicos **	48	9,7	10,0	2,2	4,9	14,2
Atendimentos de enfermagem **	48	4,4	4,5	1,1	2,0	6,5
Visitas domiciliares de ACS ***	48	26,2	26,6	3,6	19,7	33,9
Variáveis de controle						
Estimativa do % de cobertura da APS	48	69,52	69,29	4,52	58,48	75,37
Número de médicos(as) **	48	49,6	50,3	1,63	46,3	51,8
Número de enfermeiros(as) **	48	56,9	56,6	1,74	54,0	59,9
Número de ACS ^#^	48	281,4	281,7	0,8	279,9	282,8

ACS: agentes comunitários de saúde; APS: atenção primária à saúde; DP: desvio padrão.

* De agosto de 2007 a dezembro de 2015;

** Em milhares;

*** Em milhões;

^#^ De janeiro de 2016 a dezembro de 2019.

A média da estimativa do percentual de população coberta pela APS, entre 2007 e 2019, foi de 69,5%, sendo que, em dezembro de 2019, foi de 74,6%. No período, houve uma ampliação dos números de médicos de 37.230 para 51.810, de enfermeiros de 35.870 para 59.910 e de ACS de 228.250 para 282.880.

A Tabela 2 apresenta o impacto da implantação do novo sistema de informação da APS (SISAB) a partir de janeiro de 2016 nos registros de atendimentos médicos, de enfermagem e nas visitas domiciliares de ACS.

**Tabela 2:** Sumarização do impacto nos registros da atenção primária à saúde após implantação do Sistema de Informação em Saúde para a Atenção Básica (SISAB). Brasil, 2007 a 2019.

Variáveis	Média mensal (em milhões)	Acumulado (em milhões)
Atendimentos médicos		
Atual	9,66	463,47
Predição (DP)	12,86 (0,24)	617,31 (11,65)
IC95%	12,40; 13,35	595,28; 641,02
Efeito absoluto (DP)	-3,21 (0,24)	-153,85 (11,65)
IC95%	-3,70; -2,75	-177,56; -131,81
Efeito relativo (DP)	-0,25% (0,02%)	-0,25% (0,02%)
IC95%	-0,29; -0,21	-0,29; -0,21
Valor de p da área da calda posterior	0,003	
Probabilidade de efeito causal	99,997	
Atendimentos de enfermagem		
Atual	4,39	210,61
Predição (DP)	8,97 (0,25)	430,46 (12,01)
IC95%	8,47; 9,41	406,74; 451,68
Efeito absoluto (DP)	-4,58 (0,25)	-219,84 (12,01)
IC95%	-5,02; -4,09	-241,06; -196,13
Efeito relativo (DP)	-0,51% (0,03%)	-0,51% (0,03%)
IC95%	-0,56; -0,46	-0,56; -0,46
Valor de p da área da calda posterior	0,003	
Probabilidade de efeito causal	99,997	
Visitas domiciliares de ACS		
Atual	26,58	1.275,7
Predição (DP)	31,25 (0,35)	1499,9 (16,75)
IC95%	30,58; 31,86	1.467,62; 1.529,38
Efeito absoluto (DP)	-4,67 (0,35)	-224,20 (16,75)
IC95%	-5,28; -4,00	-253,68; -191,92
Efeito relativo (DP)	-0,15% (0,01%)	-0,15% (0,01%)
IC95%	-0,17; -0,13	-0,17; -0,13
Valor de p da área da calda posterior	0,003	
Probabilidade de efeito causal	99,997		

ACS: agentes comunitários de saúde; DP: desvio padrão; IC95%: intervalo de 95% de confiança.

Nota: o valor “atual” significa a média mensal de registros após a intervenção. A “predição” é a média mensal estimada pelo modelo caso a intervenção não tivesse ocorrido. O “efeito absoluto” é a diferença entre a média mensal “atual” e a “prevista” pelo modelo. O “efeito relativo” é a transformação do efeito absoluto em proporção, ou seja, qual a proporção de mudança entre a média mensal “atual” e “prevista” pelo modelo.

O valor “atual” significa a média mensal de registros após a intervenção. A “predição” é a média mensal estimada pelo modelo caso a intervenção não tivesse ocorrido. O “efeito absoluto” é a diferença “entre a média mensal “atual” e a “prevista” pelo modelo”. O “efeito relativo” é a transformação do efeito absoluto em proporção, ou seja, qual a proporção de mudança entre a média mensal “atual” e “prevista” pelo modelo.

**Figura 1:**
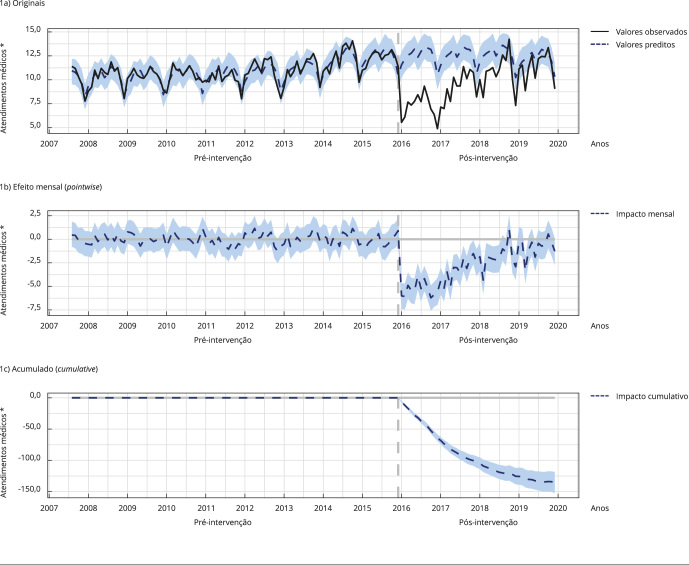
Impacto da implantação do Sistema de Informação em Saúde para a Atenção Básica (SISAB) nos registros de atendimentos médicos.

**Figura 2:**
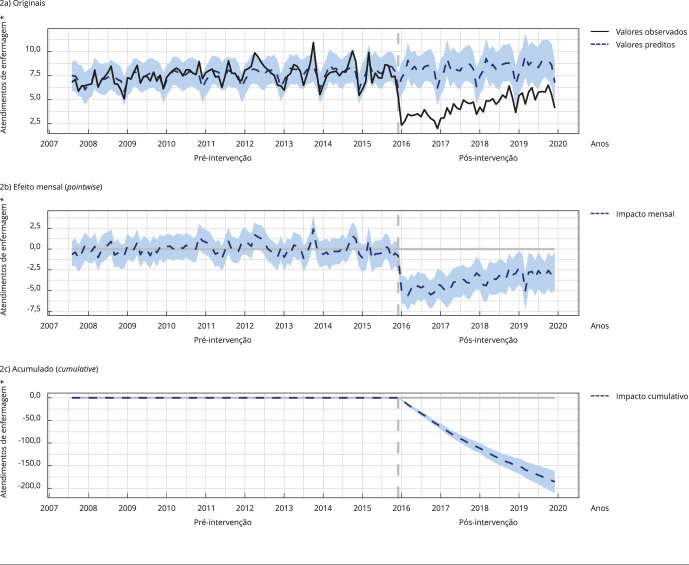
Impacto da implantação do Sistema de Informação em Saúde para a Atenção Básica (SISAB) nos registros de atendimentos de enfermagem.

As Figuras 1, 2 e 3 apresentam graficamente o impacto, demonstrando os registros originais; contendo os valores observados (linha preta) e os valores estimados pelo modelo com base nos *a priori* ou contrafactuais caso a intervenção não tivesse ocorrido (linha azul tracejada), o efeito mensal (*pointwise*), demonstrando a variação média mensal observada nos registros (linha tracejada azul) em comparação com a média estimada; e acumulado (*cumulative*) ao longo do tempo, demonstrando a soma do efeito mensal ao longo do tempo (linha azul tracejada).

Nota: as Figuras 1a, 1b e 1c apresentam graficamente o impacto, demonstrando os registros originais; contendo os valores observados (linha preta) e os valores estimados pelo modelo com base nos *a priori* ou contrafactuais caso a intervenção não tivesse ocorrido (linha azul tracejada), o efeito mensal (*pointwise*), demonstrando a variação média mensal observada nos registros (linha tracejada azul) em comparação com a média estimada; e acumulado (*cumulative*) ao longo do tempo, demonstrando a soma do efeito mensal ao longo do tempo (linha azul tracejada). * Em milhões.

Nota: as Figuras 2a, 2b e 2c apresentam graficamente o impacto, demonstrando os registros originais; contendo os valores observados (linha preta) e os valores estimados pelo modelo com base nos *a priori* ou contrafactuais caso a intervenção não tivesse ocorrido (linha azul tracejada), o efeito mensal (*pointwise*), demonstrando a variação média mensal observada nos registros (linha tracejada azul) em comparação com a média estimada; e acumulado (*cumulative*) ao longo do tempo, demonstrando a soma do efeito mensal ao longo do tempo (linha azul tracejada). * Em milhões.

**Figura 3:**
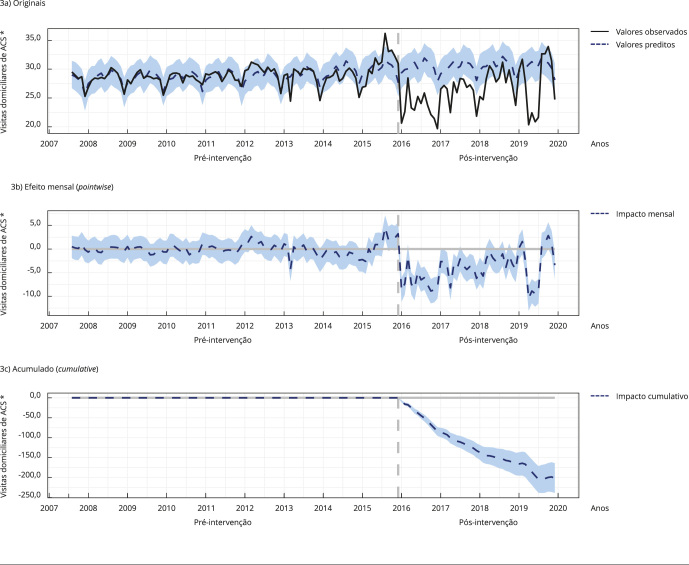
Impacto da implantação do Sistema de Informação em Saúde para a Atenção Básica (SISAB) nos registros de visitas domiciliares de agentes comunitários de saúde (ACS).

Nota: as Figuras 3a, 3b e 3c apresentam graficamente o impacto, demonstrando os registros originais; contendo os valores observados (linha preta) e os valores estimados pelo modelo com base nos *a priori* ou contrafactuais caso a intervenção não tivesse ocorrido (linha azul tracejada), o efeito mensal (*pointwise*), demonstrando a variação média mensal observada nos registros (linha tracejada azul) em comparação com a média estimada; e acumulado (*cumulative*) ao longo do tempo, demonstrando a soma do efeito mensal ao longo do tempo (linha azul tracejada). * Em milhões.

Em relação aos atendimentos médicos, durante o período de uso único do SISAB, houve, em média, 9,66 milhões de atendimentos por mês. Na ausência da implantação do SISAB, teríamos esperado uma média de 12,48 milhões ao mês. Subtrair essa previsão da resposta observada produz uma estimativa do efeito que a intervenção teve na variável resposta. Esse efeito é, em média, menos 2,82 milhões de atendimentos ao mês.

Somando os atendimentos de cada mês durante o período após o fim do uso do SIAB, houve um resultado de 463,47 milhões de atendimentos médicos. Em contrapartida, se a intervenção não tivesse ocorrido, seria esperado um montante de 598,86 milhões. Em termos relativos, houve um decréscimo de 25% nos atendimentos médicos.

Em relação aos atendimentos de enfermagem, houve, em média, 4,39 milhões de atendimentos mensais. Na ausência da implantação do SISAB, seria esperada uma média de 8,97 milhões ao mês, o que implica um efeito médio de menos 4,58 milhões de atendimentos ao mês.

Somando os atendimentos de cada mês após fim do uso do SIAB, houve um total de 210,61 milhões de atendimentos de enfermagem. Se a intervenção não tivesse ocorrido, seria esperado resultado de 430,46 milhões. Em termos relativos, houve um decréscimo de 51% nos atendimentos de enfermagem.

Quanto às visitas domiciliares de ACS, durante o período de uso único do SISAB, houve, em média, 26,58 milhões de visitas mensais. Sem a implantação, seria esperada uma média de 31,25 milhões ao mês, o que implica um efeito médio de menos 4,67 milhões de visitas ao mês.

Somando as visitas de cada mês, houve um resultado de 1,28 bilhão de visitas. Se a intervenção não tivesse ocorrido, seria esperado um total de 1,5 bilhão. Em termos relativos, houve um decréscimo de 15% no número de visitas domiciliares de ACS.

Em todos os três casos, a probabilidade de obter esse efeito por acaso é muito pequena (probabilidade bayesiana da área da cauda posterior, valor de p = 0,001). Portanto, a associação entre as variáveis é estatisticamente significativa, sugerindo uma relação causal, tanto para os atendimentos médicos e de enfermagem, quanto para as visitas domiciliares de ACS.

## Discussão

Os resultados demonstram que a mudança do SIAB para o SISAB, a partir de janeiro de 2016, impactou de forma significativa o registro de atividades da APS, provocando redução dos números de registros de atendimentos médicos, de enfermagem e de visitas domiciliares de ACS.

A consequência foi maior nos atendimentos de enfermagem, que foram reduzidos, em média, em 51%. Analisando a evolução dos atendimentos, percebe-se que, comparando-se o fim de 2019 com o início de 2016, houve aumento do número registrado de atendimentos de enfermagem da APS nos municípios brasileiros. Contudo, no fim de 2019, os valores ainda estão distantes do patamar pré-intervenção (antes do uso único do SISAB), que estava próximo de 7,5 milhões de atendimentos ao mês.

Os impactos relativos sobre os atendimentos médicos e as visitas domiciliares de ACS foram menores (-25% e -15%, respectivamente), seja na média mensal, seja no acumulado após 2016. Apesar de uma ampliação na variância dos dados (as cristas e vales na linha do tempo estão um pouco mais distantes da média, aumentando o DP e, portanto, a variância) para esses dois desfechos, percebe-se que, no fim de 2019, o número de registros está próximo do número pré-intervenção, mostrando recuperação.

Diante da ampliação ou, no mínimo, manutenção da cobertura da APS nos municípios brasileiros no período ^20^ e diante de uma escassez de evidências, é baixa a possibilidade de que essa redução de registros tenha sido consequência da diminuição da utilização dos serviços. Ao contrário, este estudo aponta indícios de que a mudança do sistema de informação gerou um conjunto de modificações no processo de trabalho da APS, com alterações principalmente na forma de registrar as ações executadas.

Duas hipóteses, discutidas entre trabalhadores, gestores e pesquisadores da área, tentam explicar esse impacto. A primeira hipótese é que a introdução do SISAB gerou uma série de desafios, para gestores e profissionais, que não foram adequadamente enfrentados. A segunda hipótese segue outra abordagem, questionando a confiabilidade dos dados do antigo SIAB.

Começando a discussão pela primeira hipótese, destaca-se o desafio chamado “fator humano”. Como bem relatado na literatura nacional e internacional ^21^^,^^22^^,^^23^^,^^24^, o fator humano é um ponto sensível na mudança de sistemas de informação, diante das possíveis resistências às mudanças organizacionais que o novo sistema pode ter gerado. Langenwalter ^25^, por exemplo, aponta como possíveis razões do insucesso da implantação de um novo sistema de informação: (a) resistência das pessoas ao novo sistema, pois estão satisfeitas com o sistema antigo e não acham necessária a implantação de outro; (b) expectativas exageradas a respeito do novo sistema; e (c) dificuldade de entender os conceitos do novo sistema.

No Brasil, alguns estudos ^14^^,^^26^^,^^27^^,^^28^ que investigaram a experiência de implantação do SISAB também identificaram resistência à mudança, relacionada à obrigatoriedade da implantação do SISAB, sem negociação prévia, e à fragilidade do processo de capacitação para o uso do novo sistema. Em um município da Bahia, que ainda realizava registro de dados de forma manual, houve relatos de resistência diante do retrabalho de preencher impressos e depois digitá-los no sistema de informação ^26^.

Além do fator humano, outro fator determinante do sucesso ou insucesso da implantação de um sistema de informação é a infraestrutura tecnológica. Schönholzer et al. ^14^, por exemplo, apontam, como causa das dificuldades de implantação do SISAB, a inadequação da infraestrutura tecnológica, incluindo a falta de equipamentos de informática e, em alguns cenários, a dificuldade de conexão à internet.

Outros fatores determinantes do grau de sucesso da implantação estão relacionados a aspectos gerenciais. No caso do Município de Camaçari (Bahia), ocorreram dificuldades na inserção dos profissionais no CNES, houve indefinição acerca de qual profissional ficaria responsável pela digitação das fichas de Cadastro Domiciliar e Individual, acumularam-se papéis e se perderam fichas, houve negligência dos profissionais no preenchimento de campos dos formulários, entre outras falhas gerenciais ^26^. Em um município do interior da Paraíba, constataram-se falta de orientação, e consequente insegurança no preenchimento das fichas, e dificuldades em atender os usuários por meio do cartão do SUS ^27^.

Em um município do Estado de São Paulo, que já passou a utilizar o Prontuário Eletrônico do Cidadão (PEC) disponível com o novo SISAB, os relatos indicam que a implantação do sistema gerou sentimentos de desapontamento e confusão nos profissionais diante das falhas ocorridas durante o processo. Houve, ainda, uma percepção dos profissionais de verticalização das tomadas de decisão, fragilidade de planejamento e insuficiência de capacitação ^14^.

O Ministério da Saúde começou a monitorar o formato de envio das informações para o SISAB a partir de abril de 2017. Nesse mês, 63,9% das unidades básicas de saúde utilizavam o sistema de registros em papel (Coleta de Dados Simplificada - CDS), 21,4% utilizavam prontuário eletrônico próprio e 14,7% usavam o PEC disponibilizado na plataforma e-SUS AB. Já em dezembro de 2019, esses números mudam para 38,2%, 26,5% e 35,3%, respectivamente, indicando uma forte ampliação da informatização das unidades do país.

Essa ampliação não foi homogênea, uma vez que os municípios informatizaram suas unidades em momentos diferentes. Esse fato pode ter gerado ainda mais dificuldade na mudança do sistema de informação, diante da transição das fichas em papel do SIAB para as planilhas eletrônicas do SISAB. Em dezembro de 2021, 19,3% das unidades básicas de saúde no país ainda utilizavam fichas em papel (CDS), impondo o trabalho de digitação aos seus funcionários.

Para finalizar a discussão da primeira hipótese - de que a introdução do SISAB gerou uma série de desafios que não foram adequadamente enfrentados -, vale acrescentar que a transição entre sistemas de informações pode ter gerado diferentes níveis de resistências entre as categorias de profissionais de saúde, o que poderia ajudar a explicar o diferente impacto, identificado neste trabalho, entre médicos, enfermeiros e ACS. Também é possível que o impacto da introdução do SISAB tenha sido diferente entre os municípios que já utilizavam e os que não utilizam o Prontuário Eletrônico.

Assim, sugere-se que novos estudos investiguem o impacto da mudança do sistema de informação da APS nas diversas categorias profissionais e em diferentes grupos de municípios, de acordo com o grau de informatização das unidades de saúde à época da implantação do SISAB.

No que concerne à segunda hipótese - a baixa fidedignidade dos dados registrados no antigo SIAB -, há relatos ^10^^,^^29^^,^^30^^,^^31^^,^^32^ de duplicação do registro de atendimento de um usuário de acordo com a condição avaliada (hipertensão, diabetes e outras), contabilização indevida dos processos diante de dados que eram sumarizados ao fim do mês de trabalho, com dificuldade de recordação e verificação das informações, perda dos registros em papel, entre outros aspectos.

O novo SISAB teria sistematizado melhor esse processo, limitando a possibilidade de duplicação, favorecendo o uso de prontuário eletrônico com o registro automático da produção de serviços imediatamente após cada atendimento ou mesmo via CDS com dados sendo “lançados” no mesmo dia de forma descentralizada pela equipe ou pelo profissional que fez a consulta.

Nessa perspectiva, o processo de registros dos dados no SISAB seria mais confiável, sendo mais fiel à realidade do cotidiano das unidades de saúde em comparação com os dados do SIAB, o que poderia justificar a redução dos registros, indicando um certo superdimensionamento de atendimentos no período pré-intervenção.

Ainda que estudos ^31^^,^^33^ abordem a qualidade dos dados no SIAB, essa segunda hipótese carece de evidências documentadas em relatórios de gestão ou publicações científicas com métodos precisos e robustos que a sustentem. É, todavia, factível, considerando-se o longo tempo de adaptação dos profissionais ao SISAB: se o problema fosse apenas de adaptação ao novo sistema, seria esperado que o número de registros retornasse ao padrão anterior em 6 a 12 meses, que é, em geral, o período de adaptação ao uso de prontuários eletrônicos em experiências internacionais ^34^^,^^35^^,^^36^. Na prática, contudo, este estudo constatou que, após quatro anos de uso, os números permaneceram menores em relação à média pré-intervenção.

É possível que as duas hipóteses sejam verdadeiras, ou seja, problemas de adaptação ao novo sistema, gerando perda de registros, e melhoria da qualidade da informação, juntos, levaram à redução dos registros de atendimentos e visitas da APS.

Além das duas hipóteses abordadas neste trabalho, o contexto de implantação da Política Nacional de Atenção Básica (PNAB) também merece ser problematizado, diante da alteração provocada pela mudança da política em 2017 e das consequentes mudanças na direcionalidade da APS no Brasil, alterando configuração, financiamento e credenciamento das equipes, além de certa redução de ACS (na comparação entre 2017 e 2019) e enfraquecimento do Núcleo de Apoio à Saúde da Família ^32^. Tal mudança de cenário e direcionalidade pode, também, ter tido algum efeito no processo de trabalho da APS, com consequência no registro de atendimentos e visitas de ACS. Uma evidência dessa afirmação é abordada por Giovanella et al. ^37^, que identificam diferenças na afirmação dos usuários em relação a ter recebido ao menos uma visita de ACS no ano (redução de 47,2% em 2013 para 38,4% em 2019), em domicílios cadastrados na APS. Essas mudanças dão ainda mais complexidade ao processo de análise, limitando os achados deste estudo, que também precisam ser contextualizados no cenário histórico diante das mudanças ocorridas na política da APS no Brasil, no período de 2016 a 2019.

## Comentários finais

Este estudo demonstrou que a mudança do SIAB para o SISAB impactou o registro de atendimentos médicos, de enfermagem e de visitas domiciliares de ACS, reduzindo significativamente seus números. Esse impacto pode ser explicado por dificuldades de adaptação de profissionais e gestores locais ao novo sistema, por limitações da infraestrutura tecnológica e, ainda, por falhas gerenciais. Subsidiariamente, pode ser explicada, também, pela melhoria da qualidade do processo de registro, com potencial redução de duplicação ou de registros indevidos.

Ressalta-se que essa experiência de mudança de sistema, vivenciada no Brasil, tem gerado desconfiança sobre a fidedignidade do registro das ações produzidas nas unidades entre profissionais, pesquisadores e gestores responsáveis pela condução das políticas de saúde no país, dificultando o processo de planejamento das políticas da APS. Este estudo ajuda a identificar estratégias para superar essa desconfiança e aumentar a fidedignidade das informações: investir nas condições de trabalho dos profissionais, incluindo a intensificação de ações de educação permanente, melhorar a infraestrutura tecnológica das unidades de APS e qualificar a gestão local.
